# Epigenetic modifications in thymic epithelial cells: an evolutionary perspective for thymus atrophy

**DOI:** 10.1186/s13148-021-01197-0

**Published:** 2021-11-24

**Authors:** Cexun Hu, Keyu Zhang, Feng Jiang, Hui Wang, Qixiang Shao

**Affiliations:** 1 Department of Immunology, School of Medicine, Jiangsu University, Zhenjiang, 212013 Jiangsu People’s Republic of China; 2 Department of Immunology, Key Laboratory of Medical Science and Laboratory Medicine of Jiangsu Province, School of Medicine, Jiangsu University, No. 301 Xuefu Road, Zhenjiang, 212013 Jiangsu People’s Republic of China; 3 Jiangsu College of Nursing, School of Medical Science and Laboratory Medicine, Huai’an, 223002 Jiangsu People’s Republic of China

**Keywords:** Thymus atrophy, Thymic epithelial cell, Epigenetic modification, Foxn1, EMT, Rejuvenation

## Abstract

**Background:**

The thymic microenvironment is mainly comprised of thymic epithelial cells, the cytokines, exosomes, surface molecules, and hormones from the cells, and plays a vital role in the development, differentiation, maturation and homeostasis of T lymphocytes. However, the thymus begins to degenerate as early as the second year of life and continues through aging in human beings, leading to a decreased output of naïve T cells, the limited TCR diversity and an expansion of monoclonal memory T cells in the periphery organs. These alternations will reduce the adaptive immune response to tumors and emerging infectious diseases, such as COVID-19, also it is easier to suffer from autoimmune diseases in older people. In the context of global aging, it is important to investigate and clarify the causes and mechanisms of thymus involution.

**Main body:**

Epigenetics include histone modification, DNA methylation, non-coding RNA effects, and chromatin remodeling. In this review, we discuss how senescent thymic epithelial cells determine and control age-related thymic atrophy, how this process is altered by epigenetic modification. How the thymus adipose influences the dysfunctions of the thymic epithelial cells, and the prospects of targeting thymic epithelial cells for the treatment of thymus atrophy.

**Conclusion:**

Epigenetic modifications are emerging as key regulators in governing the development and senescence of thymic epithelial cells. It is beneficial to re-establish effective thymopoiesis, identify the potential therapeutic strategy and rejuvenate the immune function in the elderly.

## Introduction

The thymus is the primary lymphoid organ and supports T cell development, which develops from the third endodermal pouch and the third ectodermal cleft [1]. The thymic microenvironment is comprised of non-lymphoid stromal cells that function as a unique orchestrator in the development, differentiation, and maturation of T cells and maintains the T cell-related central tolerance. Whereas the thymic stromal cells account for only 1% of the total cellularity of the thymus, which consists of thymic epithelial cells (TECs), dendritic cells, and endothelial cells [2]. And TECs are composed of cortical thymic epithelial cells (cTECs) and medullary thymic epithelial cells (mTECs), which primally sustain T cell development in different stages of thymopoiesis. Procedurally, T-cell progenitors (pro-T) originate in the bone marrow and migrate to the thymus outer cortex, then suffer initiate T-cell receptor (TCR) genes rearrangement and become T-cell precursors (pre-T). The commitment of T-cell lineage was induced by the interaction between Notch receptor, pre-T expression, and Notch ligands which expressed on TECs and thymic dendritic cells [3]. Based on the cell-surface molecule markers (CD4, CD8), T cells undergo a series of differentiation steps. In the thymic cortex, pre-T starts as CD4^–^CD8^–^ double-negative (DN) stage, which can be further subdivided based on cell-surface molecules CD25, CD44 CD117, firstly turns CD25^–^CD44^+^CD117^+^ (DN1) into CD25^–^CD44^–^CD117^–^(DN4) and then becomes CD4^+^CD8^+^ double-positive (DP). At the same time, the DP cells cease to proliferate and begin to arrange the *α*:*β* T-cell receptor genes again, which facilitates DP T cells to express diverse TCR. And then immature DP thymocytes that recognize self-peptide:self-MHC complexes receive signals for survival. Those who interact strongly or weakly with self-antigens are removed from the repertoire. This process is well-known as positive selection. Accordingly, cTECs mediate the positive selection of developing thymocytes. And then DP cells mature into single-positive (SP) CD4^+^ or CD8^+^ T cells, after that SP thymocytes migrate to the medulla. In the medulla, mTECs facilitate the elimination of self-reactive thymocytes to prevent autoimmunity (negative selection). At last, the developing T cells complete their maturation differentiation by undergoing repertoire selection. After this process, mature T cells immigrate into the peripheral blood, where they circulate and exchange continuously between the blood, lymphatic vessels, lymphoid organs, and different tissues. Therefore, TECs are the indispensable stromal components in nurturing and educating developing T lymphocytes towards maturation in the thymus [4].

However, in human beings and mice, the thymus begins to degenerate in the early phase. Thymic size and function reach their maximum during fetal and perinatal development, and then naturally undergo a chronic progressive regression, display a reduced thymic mass and cellularity with age, together with a perturbation of the normal thymic architecture and the replacement of thymus parenchyma by adipose tissue. The net outcome is a reductive emigration of naïve T cells to the periphery [5] and an increase of autoreactive T cells. Briefly, thymus atrophy leads to a weakened immune system and susceptibility to diseases, such as tumors and autoimmune diseases. Thus, clarifying its causes and the complex mechanisms is pivotal to provide an effective therapeutic tactic.

Epigenetic modification is a regular and natural phenomenon in which the changes in transcription activity and cell functionality are inheritable and reversible without altering the DNA sequence itself. In addition, transcription factors collaborate with remodeling regulators and epigenetic modifiers to institute and sustain the transcription permissive or restrictive chromatin environments. In general, epigenetics include DNA methylation, histone modification, non-coding RNA effects, and chromatin remodeling [6]. It is described that epigenetic modification plays a fine-tuning role in the development, differentiation, and senescence of TECs.

DNA methylation patterns and, in particular, the 5-methylcytosine change with age. This DNA methylation status can be used to estimate the chronological age and the risk of age-related diseases. Therefore, it is termed the ‘epigenetic clock’ [6, 7]. The effects of DNA methylation on thymus development and involution are complex. Previous studies have mainly focused on the epigenetic mechanisms involved in the development and differentiation of thymocytes under physiologic or pathologic conditions [8]. Nevertheless, TECs specific gene expression is also under epigenetic regulation that is not elaborated. The DNA methylation modification of the Aire gene in mTECs may participate in the terminal differentiation stage of the mTECs [9]. Likewise, Foxn1 gene transcription expression may be suppressed by DNA methylation. The C20 region locates at the first Foxn1 intron that is close to the foxn1 promoter in the human TECs line. The region is in the status of CpG hypermethylation that restrains the expression of Foxn1, the decline of Foxn1 level characterizes the age-related thymus involution [10].

Histone modification and chromatin remodeling contribute to stable regulation of gene expression. The nucleosome is the basic organizational structure of chromatin, which is comprised of 8 histone proteins (2 copies of H2A, H2B, H3, and H4) and DNA around them. Histone post-translational modification (methylation, acetylation, and ubiquitylation) and nucleosome positioning can regulate chromatin accessibility and vary with aging [11, 12]. Accessible chromatin regions connect with TF binding and other proteins (remodeling complexes and epigenetic modifiers) to regulate the transcriptional permissive or restrictive chromatin states in thymus development and involution [8]. The absence of polycomb repressive complex 2 (PRC2) activity that catalyzes the repressive H3K27me3 epigenetic marks compromises mTECs development and TCR diversity of thymocytes through the transcription factor Irf7 and Ascl1 [13].

The disruption and loss of heterochromatin are partially associated with ageing, which is also regulated by several forms of epigenetic modifications, including H3K9me3, H3K20me3, and heterochromatin protein 1α (HP1α). The close links between HP1α and cellular senescence are further confirmed in the mesenchymal stem cells [14]. Recent work has demonstrated that heterochromatin loss in hematopoietic stem cells also results in notable thymus involution [15].

In this review, we elucidate the important molecule involved in the TECs aging/senescence and then present the characteristics of thymic involution. Moreover, the close links between TECs senescence and epigenetic modifying net connected with thymic involution are discussed to better understand the mechanism. Finally, we summarize progress and prospects for therapeutics in thymus atrophy.

## The development and differentiation of TECs and epigenetic mechanisms

### The origin, development and biological hallmarks of TECs

TECs are the major components of thymic stromal cells. And the bipotent TEPCs are recognized as the common origin of cTECs and mTECs in the postnatal thymus [16]. Recently, bipotent progenitors are defined as CD45^−^EpCAM^+^CD205^+^IL-7^+^β5t^+^MTS24^+^, and mature cTECs are defined as EpCAM^+^Ly-51^+^CD45^−^ and keratin8. Although it is elusive on the specific mechanisms that bipotent TEPCs drive the generation of the cTECs [4, 17, 18]. Events occurring in the mTECs lineage are extensively understood. Briefly, bipotent progenitors pass through a phase when they express cTEPs phenotypic markers (CD45^−^EpCAM^+^CD205^+^β5t^+^IL-7^+^) before generating mTEPs in the adult thymus, and then mTEPs differentiates into mature mTECs. Correspondingly, mTECs are defined as EpCAM^+^UEA-1^+^CD45^−^ and keratin5. Based on cellular surface markers (CD80, CD86, MHC-II, Aire, RANK), mTECs are divided into four major subtypes, termed mTEC I-IV. These subtypes have distinct transcriptional and molecular features [4, 19]. Nevertheless, the specific function of these subsets is needed to further grope in T cell development, thymopoiesis, and thymic involution.

### Signaling pathways in the development and differentiation of TECs

Considering that the interaction between developing thymocytes and TECs contributes to thymus development, well known as thymus crosstalk. The development and differentiation of cTECs rely on the signaling from the DN and DP thymocytes whereas mTECs rely on SP thymocytes. Research progress on cTECs is not entirely clear. Therefore we focus on the signaling pathway on mTECs maturation. Firstly, the activation of the Notch signaling pathway is indispensable for the specification of the earliest mTECs lineage [20]. The deletion of Notch1 in TECs during the embryonic period contributes to the depletion of mTEPCs and a significant loss of mTECs [21]. Secondly, the NF-κB signaling pathway is also necessary for the mTECs specification and differentiation, which is governed by several members of the TNF receptor family, including receptor activator of nuclear factor κB (RANK), lymphotoxin *β* receptor (LTβR), and CD40 [22–25]. Early-stage mTECs development is driven by RANK and CD40 signals whereas late-stage mTECs terminal differentiation is driven by LTβR, underlining the importance of cross-talk between maturation thymocytes and thymic epithelial cells [26]. The establishment of self-tolerance also requires the CD40 signal in postnatal mice [24]. Thirdly, the function of mTORC1 in regulating TECs development and maturation is widely reported [27]. MTORC1 controls TECs proliferation by inhibiting the activity of autophagy. Treatment by mTORC1 inhibitor gives arise to a notable decrease of TECs and then has a high obstruction of thymocyte sub-populations differentiation and output [27]. MTORC2 suppresses the activity of the NF-κB signaling pathways and prevents the maturation of mTECs by inhibiting the expression of the RANK and LTβR [28]. At last, the Wnt signaling pathway is also irreplaceable for Foxn1 expression and TECs function, which principally runs through Wnt4- and Wnt5b-dependent signals [29].

### Epigenetic modification mechanisms involved in mTECs development and differentiation

Recently, histone deacetylase 3 (HDAC3) has been reported to govern the commitment of the mTECs lineage through an independent of the NF-κB signaling. Whereas deletion of either HDAC1 or HDAC2 contributes to a slight effect on the thymic size and TEC cellularity. Indeed, Foxn1-Cre mediated deletion of the HDAC3 locus leads to the nearly complete deficiency of mTECs. The microarray analysis indicates that promiscuous gene expression of tissue-restricted antigens (TRAs) is also dramatically damaged, which is dependent on the activity of the Aire gene. The limited transcription factors caused by HDAC3 deficiency are mostly mTECs-specific, accompanied by the improvement of a cTECs transcription program. These results suggest that HDAC3 is critical to regulating the mTECs lineage [30]. Another epigenetic regulator is the deacetylase Sirtuin 6 (SIRT6). Foxn1-Cre mediated TECs-specific SIRT6 ablation leads to the severe thymic hypoplasia caused by the impaired proliferation of mTECs but not cTECs. However, the differentiation from CD80^−^ mTECs to CD80^+^ mTECs has been specifically accelerated partially involved the enhanced NF-κB-SpiB-osteoprotegerin (OPG) negative feedback regulation pathway. What's more, the expression of TRAs and the maturation of nTreg cells (Foxp3^+^CD4^+^SP) are blocked obviously in SIRT6 cKO mice. Accordingly, H&E staining demonstrates more severe lymphocytic infiltration in multiple organs, including the kidney, lung, and liver. It is suggested that the establishment of central immune tolerance is disrupted after SIRT6 deletion [31]. Collectively, the expression of epigenetic regulator SIRT6 in TECs is responsible for the development and differentiation of the mTECs lines (Fig. 2). Intriguingly, the progressive loss of MHC-II and Aire expression is recently shown in mature mTECs lines [32], evidence if epigenetic regulation controls this process remains poorly understood.

TECs development and differentiation are also controlled by a handful of genes. Inactivation of them is also partially responsible for the TECs senescence. The initial stages of TEC development are regulated by the forehead box protein N1 (Foxn1), a pivotal transcription factor expressed in the TECs. It is required for all stages of TECs development and differentiation both in the fetal and adult thymus [33]. Correspondingly, Foxn1 deficiency results in apparently thymus atrophy. Intriguingly, Foxn1 expression is partly left to bone morphogenetic proteins 4 (BMP4), and BMP4 belongs to the TGF-*β* family and mainly participates in maintaining the stemness of epithelial progenitors [34, 35].

Earlier studies have unveiled that microRNAs (miRNAs) are important for governing TECs differentiation, development, proliferation, apoptosis, and/or senescence, and the changes eventually result in thymic microenvironment disorganization and damages thymocytes output [36]. It is best illustrated by the inactivation of Dicer, the key enzyme in miRNA biogenesis [37]. Such as miR-181b-5p is lessened in the Dicer-silenced mTECs line compared with the control. It is interesting to note that miR-181b-5p can impact the adhesion process between mTECs and single-positive CD4 and CD8 thymocytes, a principal step of negative selection, and repress the surface marker CD80 expression in mTECs potentially by targeting genes Tnc, Lgals3bp, Lgals9, and CD47 [38]. Additionally, miR-449a can prompt TEPCs differentiation and mature into mTECs in vitro by targeting the epigenetic regulator Satb2 which is well known to orchestrate the higher-order chromatin structure. Besides, introduction of miR-449 directly diminishes the expression of Satb2, eventually modifying the mTECs program [39]. Its function can be partially compensated by miR-34a, a miRNA sharing a similar seed sequence with miR-449a [39].

## The characteristics of thymic involution

Thymus deterioration begins in the early postnatal life and becomes more pronounced during puberty. It affects the adaptive immune responses significantly but to a lesser extent the innate immune system [40]. It can be divided into physiological thymus atrophy (age-related thymic involution) and pathological atrophy (acute thymic involution) according to different causes.

### Acute thymic involution

The thymus undergoes transient and reversible deterioration, namely, acute thymic involution (ATI). It is characterized as the resilient capacity after the removal of the causative factors [41]. ATI is receiving widespread and ongoing attention, which is the result of infectious agents, such as SARS-CoV-2 [42], Mycobacterium tuberculosis [43], Trypanosoma cruzi [44], Paracoccidioides brasiliensis [45], immune suppressor [46], radiotherapy, chemotherapy [47], glucocorticoid [48]. ATI may be also contributed to the change of physiology, hormones, emotion and gestation period [49]. TECs recognize and respond to various pathogen-related molecular models (PAMPs) [50] and then release the proinflammatory cytokines (IL-6, TNF-α, IL-1α and IL-1β) in the case of bacterial infection through Toll-like receptors (TLRs). The transcriptional level of proinflammatory cytokine IL-6 is dramatically elevated in endotoxin-induced ATI compared to controls [46]. In brief, the activation of TLR4 induces a growing cascade of the pro-inflammatory cytokines via the hypothalamus–pituitary–adrenal (HPA) axis [41], the secretory cytokines can enhance the senescence and damage the thymic microenvironment through autocrine and paracrine signalling [51].

### Age-related thymic involution

Age-related thymic involution is an evolutionarily ancient and conservative process that occurs in almost all vertebrates [52]. Of note, cell cycle-related gene expression and transcription factor E2F3 activity are initially attenuated in mTECs as early as 2 weeks after birth, whereas pro-inflammatory response, chemokines, and cytokines appeared a strong upregulation during the process of thymic involution [53]. In addition, peripheral T cells go back to the thymus and facilitate thymic involution via destroying the homeostasis of the thymic microenvironment [54]. Different from young mice, the stromal organization of the aged thymus is disordered through HE staining, displaying a damaged thymic mass, a perturbation of the normal thymic architecture, the replacement of thymus parenchyma by adipose tissue and the formation of Hassall's corpuscles. Hassall's corpuscles are comprised of terminally differentiated mTECs with a characteristic swirled epithelial structure [55]. Those alterations are at least in part responsible for the augmenting susceptibility and mortality to infectious diseases, decreased responses to vaccination, and an increased propensity for cancers and autoimmune diseases in older people [56–60]. Moreover, lessened recruitment of the pro-T cells may be an elementary explanation for the limited thymopoiesis with age. Particularly important is that the destruction of the microenvironmental compartment is considered the main reason for decreasing the output of the naïve T cells [2]. Thymic involution represents a weakened adaptive immune response and susceptibility to disease, but the underlying mechanisms are not yet fully understood. Thus, we summarize the molecular mechanism of epigenetic modifying and transcription factors related to the thymus atrophy, as well as the process of thymus adipose transformation.

## Epigenetic modification of TECs in thymus atrophy

### The features and epigenetics of senescent TECs

**Fig. 1:**
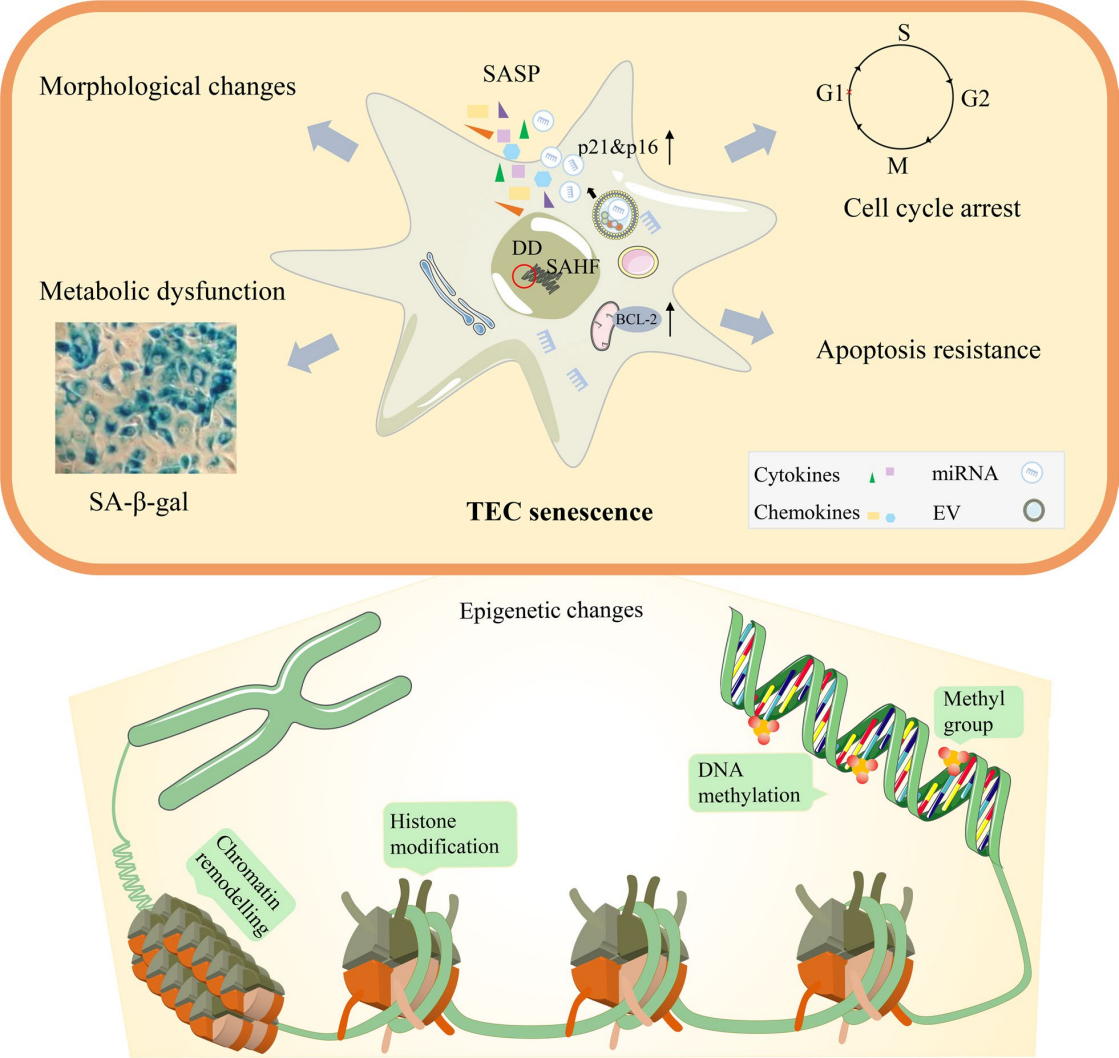
The features and epigenetic changes of TEC senescence. Cellular senescence is characterized by abnormal cell enlargement, cell cycle arrest (by upregulation of p21 & p16 cell cycle inhibitors), resistance to apoptosis (by upregulation of the BCL-2 family), SASP (by upregulation of cytokines, chemokines, extracellular vesicles), and SAHF, metabolic dysfunction (the positive of SA-β-gal staining). Epigenetic changes include DNA methylation, histone modifications and chromatin remodelling, which together play a fine-tuning role in the development, differentiation, and senescence of TECs. SASP, senescence-associated secretory phenotype; SAHF, senescence-associated heterochromatin foci; SA-β-gal, senescence-associated-β-galactosidase; DD, DNA damage; EV, extracellular vesicles

Because of the deficiency of single, universal biomarkers, senescent cells or tissue samples is hard to identify. At present, the recognized characteristics of senescent cells are the increased expression of cell cyclin-dependent kinase inhibitors p16 and p21, the anti-apoptotic BCL-2 protein family (BCL-2, BCL-W, BCL-XL), and the senescence-associated secretory phenotype (SASP) comprising cytokines, chemokines, extracellular vesicles (EVs), etc. [51, 61]. Besides, senescent cells are usually flat morphology, the abnormally enlarged together with the deficiency capacity for DNA replication, accompanied by the positive of senescence-associated-β-galactosidase (SA-β-gal) staining (Fig. 1).

Cellular senescence is usually responsible for organ aging and disease. And aging is deemed to be at least partially associated with the loss of heterochromatin that is attributed to epigenetic changes. Such as Jumonji domain-containing protein 3 (Jmjd3), also called Kdm6b, is a histone H3K27 demethylase. It plays a core role in cellular senescence by demethylating H3K27me3 sites and removal of the repressive polycomb group (PcG) proteins from the promoter sequences of senescence genes, such as p53 and p16 [62]. Indeed, Jmjd3 is directly induced by LPS via the NF-κB signaling pathway [63], suggesting that Jmjd3 lies at the nexus of inflammation and senescence. Moreover, the expression of TRA genes also requires the activity of Jmjd3. Foxn1-Cre mediated deletion of Jmjd3 in TECs is found to result in the raised levels of H3K27me3 at the promoter regions of TRAs, such as Cxcl3, Mefv, Dcstamp, Kcnd3, which hampers the promiscuous gene expression of TRAs and eventually results in the emergence of the autoimmune disease. Intriguingly, the elimination of Jmjd3 is partly responsible for the promoting apoptosis of mTECs through H3K27me3 repressing the expression of Bcl-2 [64].

### Epigenetic modification of non-coding RNAs

Comprehensive application of the high-throughput transcriptome sequencing technologies and bioinformatics allows us to better understand the whole transcriptional change, predict functional miRNA:mRNA targetpairs, and investigate the underlying molecular mechanism [65]. The biological functions of miRNAs in the development of thymocytes are comprehensively reviewed by Xu and his colleagues [66]. Nevertheless, the changes and molecular mechanisms of miRNAs in TECs with age are not clear. Giving the central role of miRNA in the TECs lineages, we summarize data regarding the role of microRNAs, targeting genes, and signaling pathways involved in TEC biology. Global transcriptome and miRNAome profiling in TECs derived from 1-and 3-month-old BALB/C mice displayed that miR-205-5p, miR-199b-5p, miR-183-5p, and miR-200b-3p are significantly upregulated in the 3-month-old mice [67]. Among them, miR-205 is epithelial cell-specific, well-known as a biomarker of mTECs [68, 69]. And miR-205-5p mimics dramatically block cell cycle in the G1 phase and interrupts the proliferation of TECs by modulating the Wnt signaling pathway and the transcription factor AP-2α [70]. Interestingly, miR-199b-5p is found to govern the expression of Wnt4 and c-myc and potentiate the proliferation of the mTECs through directly targeting the frizzled receptor 6 (Fzd6) [71]. Remarkably, miR-183 and miR-200b are closely associated with transcription factor (Meis1) which is enriched in immature TECs expression and is downregulated with age. Meis1 inactivation is not beneficial to the proliferation, differentiation, and maturation of postnatal TECs [72]. Therefore, those disordered miRNAs may be considered as the potential therapeutic targets of thymus involution in mTECs. Recently, independent miRNA sequencing (RNA-seq) analyses from different laboratories highlight the miRNA expression changes in mTECs between the 2-month-old (young) and the 20-month-old (aged) C57BL/6 mice. And 13 candidates were confirmed to be attenuated with age by quantitative polymerase chain reaction (qPCR). The variation is consistent with thymic weight loss and diminished thymopoiesis, implicating that miRNAs play a fine-tuning role in age-related thymic atrophy [73]. Those miRNAs include miR-155, miR-146a, miR-194, miR-192, miR-19a, miR-19b, miR-181a and miR-181b. The role of miR-155 was reviewed in detail and involved in the Treg cells development [74]. Moreover, miR-155 can augment the maturation of mTECs via modulating the TGF-β signaling pathway [75]. Remarkably, a negative feedback regulation loop was unveiled between miR-146a and NF-κB signaling pathway via combining to TNF receptor-associated factor 6 (TRAF6) [76]. Moreover, miR-146a-5p expression is weakened in primary thymic stromal cells in D-galactose induced premature senescence model, while the introduction of miR-146a-5p could alleviate the cell cycle arrest and suppress the senescence process via lowering TRAF6 protein [77]. Therefore, it may play a critical role in decaying stress and inflammatory thymic atrophy. Besides, upregulated miR-195a-5p, in the aged TECs, can also hamper the proliferation of mTECs by directly targeting Smad7 that negatively regulating the TGF-β signaling pathway [78]. In contrast, how other miRNAs supervise thymic involution remains to be determined (Table 1).

**Table 1:** List of TECs-associated miRNAs

miRNA	Expression	Target and/or regulators	Signaling pathways	Functions	References
miR-205-5p	Up	Fa2h/AP-2α, Foxn1	Wnt	miR-205-5p could block cell cycle and interrupt the proliferation of TECs	[70]
miR-199b-5p	Up	Fzd6/c-myc	Wnt	miR-199b-5p could enhance the proliferation of the mTECs through directly targeting the frizzled receptor 6 (Fzd6)	[71]
miR-183-5p miR-200b-3p	Up	Meis1	–	miR-183 and miR-200b could regulate the proliferation, differentiation, and maturation of postnatal TECs	[72]
miR-155	Down	–	TGF-β	miR-155 could facilitate the maturation of mTECs via modulating the TGF-β signaling pathway	[75]
miR-146a-5p	Down	TRAF6	NF-κB	Overexpression of miR-146a-5p could alleviate the cell cycle arrest and suppress the senescence process via downregulated the TRAF6 protein levels	[77]
miR-195a-5p	Up	Smad7	TGF-β	miR-195a-5p could hamper the proliferation of mTECs by directly targeting Smad7 that negatively regulating the TGF-β signaling pathway	[78]
miR-181a-5p	Down	YY1, Smad3, c-myc	TGF-β/NF-κB	miR-181a-5p could regulate the proliferation of mTECs in vitro possibly by the phosphorylation of Smad3 which plays a key role in the TGF-β signaling	[80]
miR-181b-5p	Down	Tnc, Lgals3bp, Lgals9, Cd47	–	miR-181b-5p could impact the adhesion process between mTECs and single-positive CD4 and CD8 thymocytes and repress the surface marker expression of CD80 in mTECs	[38]
miR-449a	–	SATB2	–	miR-449a can prompt TEPC differentiation and mature into mTECs in vitro by targeting the epigenetic regulator Satb2	[39]
miR-125a-5p	Up	Foxn1	–	miR-125a-5p mimics have a fine-tuning role for suppression expression of Foxn1 in murine TECs Z210 cells via directly target 3’UTR of Foxn1	[89]

Of note, the expression level of the miR-181 family is 20-fold higher in the thymus than in the brain [79] but decreases with age, a direct correlation is observed between miRNA-181a-5p and proliferation of mTECs in vitro possibly by regulating the phosphorylation of Smad3 which plays a key role in the TGF-β signaling [80]. Whereas, removal of miR-181a1 and miR-181b1 in TECs seems to be only associated with the lower number of cTECs in *vivo*, but not affected the positive selection and expedited thymic involution [81], mirroring that the function of the miR-181 family is possibly implemented overlapping functions (Table 1).

Recently, circular RNAs (circRNAs) have been reported to be involved in biological processes through the post-transcriptional modification of miRNAs. Therefore, circRNA expression profiles among mice thymuses at 6 weeks and 3 months are analyzed for the first time by the RNA-sequencing technique to understand the molecular mechanisms of thymic involution. Indeed, the differential expression genes in 3 months of the thymus are primarily associated with fatty acid biosynthesis and metabolism, which are thought to be linked to the thymus regression [82]. However, the expression of circRNAs in senescent TECs is not clarified and needs to be further investigated. Furthermore, several circRNAs are identified as the sponges of miRNAs and may present antiviral activity in Zika virus-infected human TECs, for instance, hsa-miR-92a-1-5p possibly regulated by hsa-circ-0076612 may act as the antiviral immunity against ZIKV-infected TEC [83].

Long non-coding RNAs (lncRNAs) are recently discovered as the epigenetic regulator in stress-induced thymic involution. After 17-estradiol (E2) stimulation, the differentially expressed lncRNAs are predicted to be mainly involved in cell cycle and apoptosis in mTECs, like Gm26870 and TCONS_00067179 [84]. Correspondingly, lncRNAs expression is also widely affected after ovariectomy or orchiectomy, like lnc191945, lnc180466 and lnc186500 [85]. Taken together, those studies suggest that lncRNA may govern the proliferation of TECs and physiological aging processes by regulating the expression of miRNA or transcription factors.

The studies that ncRNAs regulate TECs development and their expression changes in senescent TECs will urge us to better understand the process of thymus development and provide a new cognition to thymus involution.

### Epigenetic modification of transcription factor

#### Foxn1

The thymic function requires extensive cross-talk between developing T cells and thymic epithelium. Nevertheless, the expression of Foxn1 in TECs is weakened with age, which leads to typical age-related thymic involution [86]. Introduction of Foxn1 in aged mice improves thymopoiesis and then attenuates the clinical symptoms [87]. Remarkably, the first intron of Foxn1 is reported to have the activity of the cis-regulatory element. No apparent thymus is exhibited when Foxn1 deleted, thus contributing to a complete arrest of TECs maturation and thymic dysfunction [88].

Recently, a handful of miRNAs are unveiled to regulate Foxn1 expression. The miRNA expression profile from 2- and 21-months-old mice TECs have disclosed that miR-125a-5p, miR-342-3p, miR-6931-5p, and miR-320-5p are confirmed to be upregulated in the aged thymus, whereas the expression levels of miR-18a-5p and miR-466f are downregulated. Remarkable, miR-125a-5p mimics have a fine-tuning role for suppression expression of Foxn1 in murine TECs Z210 cells in a dose-dependent manner via directly target 3’UTR of Foxn1 [89]. Given its 3 targeting sites in Foxn 1 3’UTR, miR-320-5p is most likely another miRNA regulating the expression of the Foxn1 [89]. In addition, miR-22 directly suppresses the activity of transcription factor Foxn1 and accelerates hair follicle apoptosis in hair keratinocytes [90]. Considered these similar functions in the skin, a rational hypothesis is that miR-22 is likely to supervise the development of TECs via governing the Foxn1 expression [90]. In contrast, miR-205 is reported to maintain the expression of Foxn1 in the condition of stress [91].

Furthermore, Foxn1 recognizes the target genes to control the TECs development via a specific 5-bp forkhead-like (FHL) DNA motif (GACGC) [92]. Experiments in the Foxn1 mutation mice have manifested that some regions influence the DNA-binding of Foxn1 [93]. Interestingly, the C-terminal 175-acidic amino acids are associated with the phenotype of the athymia and nude in mice [94]. Besides, the C-terminal region of Foxn1 is essential for DNA binding [92]. However, the 5-methylcytosine at the CpG within the FHL motif is bound loosely together than the non-methylated version [92]. Considering the attenuation of the DNA methylation with age, taken together, suggesting that this will eventually contribute to thymic involution and even immunosenescence.

**Fig. 2:**
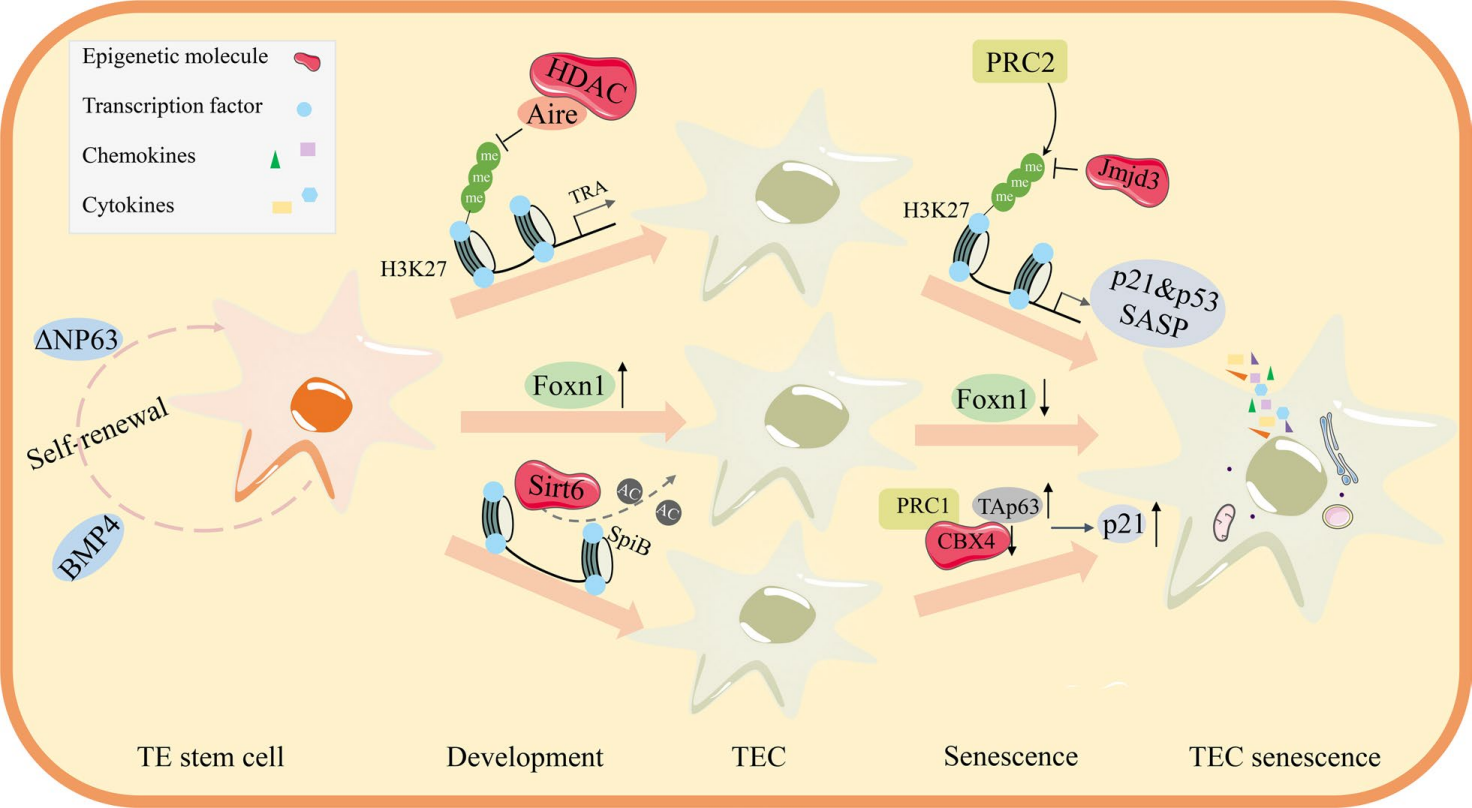
Epigenetic molecules that regulate the origin, development, and senescence of TECs. Expression of ΔNp63 and Bmp4 could maintain the stemness of epithelial progenitor cells for self-renewal and replenish the loss of mature TECs. The transcription factor Foxn1 is under multiple epigenetic modifications, which is the master regulator during thymus development. Besides, SIRT6 has been identified as a molecular switch necessary to the mTEC development and differentiation via repressed SpiB activity. Aire exerts the repressive function by enrichment of H3K27me3 to maintain the proper TRA expression. However, Foxn1 expression is reductive with age, resulting in TECs senescence associated with an increase in TAp63, which is consistent with the diminishment of the polycomb repressive complexes 1 (CBX4). Jmjd3 lies at the nexus of inflammation and senescence by demethylating H3K27me3 sites and releasing the repressive polycomb group (PcG) proteins from the promoter sequences of senescence genes, such as p53/p21, cytokines, and chemokines. Ultimately, the thymus undergoes aging. HDAC, histone deacetylase; Sirt6, sirtuin 6; Jmjd3, Jumonji domain-containing protein 3; SASP, senescence-associated secretory phenotype; TF, transcription factor

To some extent, the Foxn1 function is also dependent on the activator of the transcriptional regulators, such as chromobox homolog 4 (Cbx4) and embryonic ectoderm development (Eed). Polycomb group (PcG) proteins are assembled into a variety of compounds, and the two best well-known forms are polycomb repressive complexes 1 and 2 (PRC1 and PRC2). Cbx4, the PRC1 component, is also highly expressed in the TECs. Previous studies have revealed that Cbx4 may be a downstream factor of p63 since an obvious accumulation of p63^+^ cells in Cbx4 deficient TECs, which unveils a damaged proliferation capacity [95] (Fig. 2). This hypothesis is further confirmed in the epidermis [96]. Recently, Cbx4 deficiency is reported to lead to premature cellular senescence in the human mesenchymal stem cell [97]. However, additional analyses will be required to investigate the involvement of the p63/Cbx4 network in age-related thymic involution. Moreover, when the Eed is deleted, a core component of the PRC2, the proliferation, and differentiation of the TECs are in severe disorder [98].

#### p63

Conditional knockout of Foxn1 in the thymus of the early middle age fx/fx-uCreER^T^ animals also contributed to the ascending expression of TAp63, one of the transcription factor p63 production containing an N-terminal transactivation domain. TAp63 is co-localized with p21 and significantly associated with thymic involution, which is expressed in the TECs rather than in the thymocytes [99]. P21 is a cyclin-dependent kinase inhibitor involved in cell cycle progression, DNA repair, and inhibition of apoptosis [100]. ΔNp63 does not contain an N-terminal, which was mainly to maintain the stemness of epithelial progenitor cells. When deleted Foxn1, the mature TECs proliferation was suppressed, along with the ascending expression of TAp63 and SA-β-gal^+^. Hence, the p63-foxn1 axis may be a rational answer to regulating the TECs progenitor pool during the thymic aging [99]. Furthermore, the details that some miRNAs governed the p63 gene expression had been described in skin epithelial cells [101, 102]. Many similarities between TECs and skin epithelial cells imply further experiments should be required to confirm the senescence effect of some miRNAs by targeting the TAp63 gene. Undoubtedly, it is also a new treatment strategy for thymus rejuvenation. Impressively, miR-205 is an epithelial-related miRNA expressed especially in the TECs. miR-205 and the transcriptional factor ΔNp63 are reported to form a positive regulatory feedback loop [103] to promote the expression of Foxn1 in the TECs [91] (Fig. 2).

### Epigenetic modification of thymic adipogenesis

**Fig. 3:**
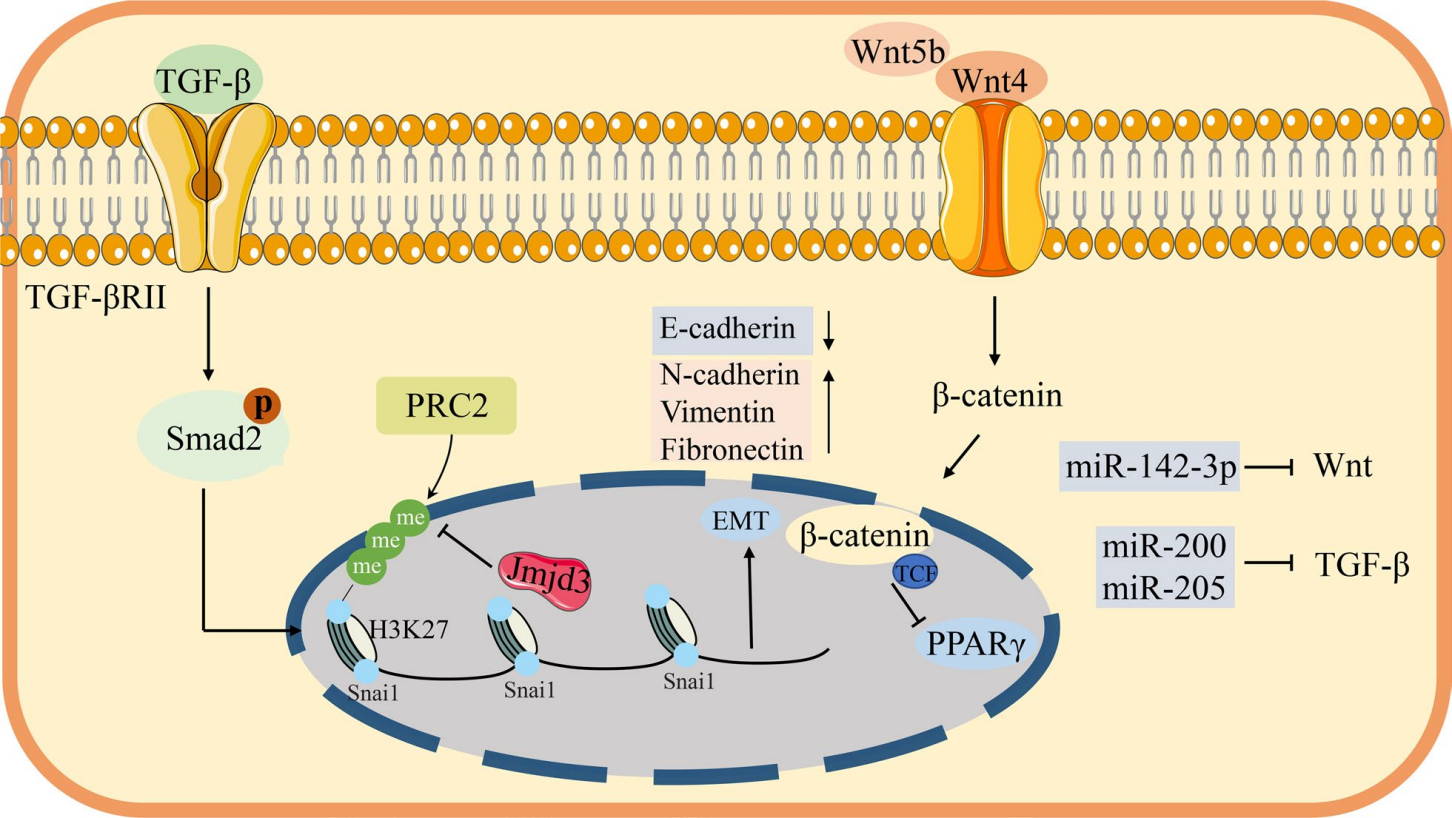
Model of thymic epithelial cell mesenchymal transition. Activation of the TGF-β signaling pathway contributes to the upregulation of mesenchymal markers by phosphorylating smad protein, such as N-cadherin, vimentin, and fibronectin, the downregulation of the epithelial markers (E-cadherin). Moreover, histone demethylase Jmjd3 can promote the formation of EMT via suppressing the activity of H3K27me3. The Wnt signaling pathway can hinder the age-related thymic adipogenesis by suppressing PPARγ expression. EMT, epithelial-mesenchymal transition; PPARγ, peroxisome proliferators-activated receptors

Thymic adipogenesis is recognized as a notable feature of thymic involution. The thymic stromal space shrinks to be replaced by adipose tissue during aging. Previous studies have demonstrated that adipocytes are not inert cells and can secrete distinct hormones and cytokines that influence the thymic microenvironment [104]. Particularly important is that the provenance of ectopic thymic adipocytes remains an unsolved puzzle. A hypothesized mechanism is the adipocyte ‘infiltration’ within perivascular space (PVS). Briefly, PDGFR-β^+^ mesenchymal cells, perivascular cells and pericytes derived from the vasculature have the potential to differentiate into adipocyte lineage through the activation of peroxisome proliferators-activated receptors (PPARγ) [105–107], but more powerful evidence is needed to be formally verified. Notably, earlier studies over the past few years have demonstrated that thymic atrophy is correlated with the adipogenesis transformation of TECs [108]. Though the mechanisms are not fully clear, a reliable version is that first epithelial-mesenchymal transition (EMT) occurs followed by subsequent transdifferentiate into preadipocytes, accompanied by the activation of the TGF-β signaling pathway in TECs. On the contrary, lacking TGF-βRII expression in mice TECs could alleviate the physiological process of thymic aging, suggesting that the TGF-β signaling pathway plays a key role in accelerating the process of thymic adipose involution [109]. Recently, Chen and his colleagues firstly elaborate the EMT mechanism that the activation of the TGF-β signaling pathway degrades the E-cadherin protein by mediating the phosphorylation of Src kinase, and then reduces the expression of E-cadherin through the p-Smad2/FoxC2 axis in TECs [110]. Interestingly, the miR-200 family and miR-205 are extensively analyzed to hamper the activation of TGF-β and prevent the TGF-β induced EMT [111] (Fig. 3).

Lamina-associated polypeptide 2alpha (LAP2α) is another extremely important protein during the thymic adipogenesis process. LAP2α is upregulated with age in TECs. It rules positively PPARγ expression that orchestrates the TECs transdifferentiation into adipocytes [112]. And adipogenic processes are also reported to be associated with the Wnt signal pathway [113–115]. The canonical Wnt signaling pathway is important for the early development of thymocytes, specifically in the development of the DN1 stage [116]. Notably, the Wnt signaling pathway was restrained in the aged thymic tissue, while additional Wnt4 protein secretion can suppress PPARγ expression, suggesting the Wnt signaling pathway is also related closely to age-related thymic adipogenesis [117]. Moreover, gene expression profiling from the elder to young human thymic samples had demonstrated that the expression of Wnt inhibitors (PPARγ, C/EBPα, and sFRP1) are significantly upregulated, whereas Wnt-related transcription factor (TCF7, TCF9, TCF12) expression are downregulated [117]. In line with previous data, miR-142-3p is reported to negatively modulate the Wnt signaling pathway and blocks the cell cycle via directly suppressing the expression of β-catenin [118], and miR-27b has been displayed to suppress the PPARγ activity and activate the Wnt signaling, thereby hindering the adipogenesis process [119–122]. Intriguingly, Wnt4 can promote mTECs proliferation by directly prompting the Foxn1 transcriptional level [123] and prevent Glucocorticoid-induced thymic senescence [124]. Taken together, TECs structure disorder [125], even the damage of the thymic microenvironment, may attribute to the attenuation of the Wnt signaling pathway.

Importantly, histone demethylase Jmjd3 also plays an indispensable role in the TGF-β induced EMT. Indeed, knocking down of Jmjd3 is found to restrain the expression of mesenchymal markers induced by TGF-β in epithelial cells, such as N-cadherin, vimentin, and fibronectin. In contrast, ChIP assays demonstrate that overexpression of Jmjd3 can promote the formation of EMT via suppressing the activity of H3K27me3 on the promoter of transcription factor Snai1. In brief, emerging of EMT is partly attributed to the activity of the TGF-β-Jmjd3-Snai1-VIM signaling axis [126] (Fig. 3).

### Epigenetics modification of tissue-restricted self-antigens expression in TECs

The promiscuous gene expression (PGE) of tissue-restricted antigens (TRAs) depends for the most part on the transcriptional factor the autoimmune regulator (Aire). This process involves the clearance of autoreactive T cells and the establishment of immune tolerance. But the expression of TRAs is diminished with age in the mTECs [127], in line with the augmented susceptibility of autoimmune diseases, implying that an underlying relevance between thymic atrophy and the morbidity of autoimmunity. Aire is bifunctional and harbors both permissive and repressive functions. Aire promotes the PGE of TRAs which are normally expressed in peripheral tissues. It also exerts its repressive function that restricts chromatin accessibility to inhibit the transcriptional duration and amplitude of TRAs, which may provide an underlying insight for the proper levels that are sufficient for immune tolerance, but above those that could be harmful [128]. It is consistent with the reported enrichment of H3K27me3 [129] and the lessened H3K4me3 levels of promoter-associated chromatin modification [130]. Conversely, Previous studies have unveiled that Aire and its partners regulate the expression of repressive chromatin of Aire-dependent TRA genes [131]. In general, Aire recognizes methylation marks at the histone H3 tail and directly interacts with chromatin through its C-terminus PHD. Likewise, the transcriptional activity of the Aire itself is also controlled by the HDAC1-HDAC2/SIN3A deacetylases complexes which may also influence the nucleus localization of Aire [132] (Fig. 2). In addition, thymic exosomes are nanosized, membranous vesicles. They are released by human TECs and attracted attention because of carrying TRAs, MHCII, and miRNAs [133], hence participated in the intercellular information exchange necessary for the maturation of the thymocytes and the formation of Treg cells [134]. Intriguingly, exosomes extracted from the serum of young mice are found to rejuvenate the expression of Aire in elderly mice, which could trigger efficient presentation of self-antigens in the thymus and reduce the production of autoreactive T cells [135]. Besides, combining deletion of NF-κB2 and Bcl-3 is responsible for severe autoimmune disease, which disturbs the negative selection process of the autoreactive T cells [136].

### Epigenetic modification of sex-steroid hormones related to acute thymic involution

Acute thymic involution usually occurs during puberty and pregnancy. Giving the fact of the ascending synthesis and secretion of sex-steroid hormones during this period in human beings. A link was found between physiological thymus involution and the release of sex hormones at puberty [137, 138]. Thymic volume dwindles significantly after puberty when sex steroid levels are augmented, especially androgens and estrogens, and large doses of sex steroids can lead to thymic regression, indicating thymus atrophy is negatively correlated with sex steroid levels [49]. Moreover, androgen receptors (AR) and estrogen receptors (ER) are highly expressed in the TECs [138]. Intriguingly, profiling analysis of mTEC1 treated with 17β-estradiol (E2) revealed that mTEC1s are significantly blocked in the S and G2/M phase, and the proliferation and viability are extensively damaged. Conversely, castration may briefly recover the function of the thymus in the older rodents relative to the young. Whereas growth hormone (GH) and insulin-like growth factor-I (IGF-I) are considered as the director of thymus atrophy as they may facilitate cell proliferation [49, 139]. Surprisingly, miR205HG is a long noncoding RNA that was able to promote the secretion of GH by enhancing the transcriptional activity of Pit1 and binding to the protein [140]. Furthermore, recent study has clearly shown that RANK in mTECs drives the development of thymic Treg cells after progesterone stimulation, which can reduce the incidence of miscarriage and prevent gestational diabetes [141].

## Looks forward to potentially therapeutic strategy for TECs senescence

Rejuvenation of immune function is of great clinical significance. Over the past decades, many works have been devoted to TECs regeneration. Given the core role of Foxn1 in TECs development and homeostasis, the therapeutic strategy based on the Foxn1-TECs axis is reasonable for the rejuvenation of age-related thymic involution. Indeed, Foxn1 overexpression is sufficient to drive thymus regeneration by directly promoting the proliferation of progenitor TECs and restoring the gene expression required for TECs function [142]. Another approach of thymic regeneration that has been identified involves the conversion of Foxn1-expressed mouse embryonic fibroblast into induced TECs (iTECs), which is conducive to ectopic thymus generation [143]. However, because of the existence of the aged thymus, the continuous releasing of self-reactive T cells inevitably promotes inflammation. Correspondingly, an intrathymic injection of freshly isolated newborn TECs for thymus rejuvenation had been proved to be infeasible, and how to collect and separate fresh neonatal TECs became an unsolvable problem. Accordingly, it is attractive to engraft directly iTECs into the aged thymus in an intrathymic injection manner. Importantly, these iTECs were indicated to enable to the generation of functional thymic tissue [144]. Furthermore, exosomes from the young serum have been manifested to facilitate the aged thymic microstructure and rejuvenate thymic atrophy through upregulating Foxn1 expression. Intriguingly, proinflammatory factor levels in the periphery of aged mice, such as IL-6, IL-1β, TNF-a, display a significant reduction after treatment with exosomes [135].

In particular, rejuvenation strategies based on TECs regeneration by cytokines have been extensively studied. One of the potential pathways of TECs regeneration is fibroblast growth factor 7 (Fgf7), also known as keratinocyte growth factor (KGF), an epithelial cell-specific growth factor [145], which actives the fibroblast growth factor receptor-2 (FgfR2-IIIb) expressed on TECs and facilitates directly the proliferation and differentiation of TECs [146]. Fgf21 induced by caloric restriction (CR) is found to delay age-related thymic atrophy. Indeed, Fgf21tg mice that overexpressed Fgf21 exhibit a close link to the cTECs function, but not the mTECs, along with thymic microenvironment improvement [147]. Another pathway is IL-22, secreted by innate lymphoid cells and Th17 cells in the thymus, which is involved in acute damage restoration [148]. IL-22 is proven to promote thymic recovery by enhancing TECs proliferation and survival capacity after total body irradiation [149]. Specifically, recent studies have reported that several important factors, such as Foxn1, Aire, and KGF, are upregulated in TECs and eventually promoted TECs rejuvenation via the IL-22/Stat3/Mcl-1 pathway [150]. Of note, several other factors are reported to be beneficial to TECs rejuvenation, including thymic endothelial cells-derived BMP4 [151], and IGF-1 [152].

## Conclusions

Despite the mechanisms of thymic involution remain poorly defined, accordingly, future research should focus on the divergent fates of certain TECs precursors and elucidation the underlying molecular mechanisms [153]. Due to the heterogeneity of TECs, the recently developed single-cell sequencing technology was extensively applied, it offers promising approaches to map the different expression gene profiles and investigate the underlying molecular mechanism of the TECs to be a reality at the single-cell level. As we focus on improving life quality, research on re-establishing effective thymopoiesis is of paramount importance. We hope this review will help to provide updated insights into the current understanding of thymic atrophy and facilitate the identification of potential candidates for therapeutic targeting.

## Data Availability

All the information is included in this manuscript.

## Abbreviations

TECs: Thymic epithelial cells
cTECs: Cortical thymic epithelial cells
mTECs: Medullary thymic epithelial cells
pro-T: T-cell progenitors
TCR: T-cell receptor
pre-T: T-cell precursors
DN: Double-negative
DP: Double-positive
Aire: Autoimmune regulator
PGE: Promiscuous gene expression
TRAs: Tissue-restricted antigens
RANK: Receptor activator of nuclear factor κB
LTβR: Lymphotoxin *β* receptor
HDAC3: Histone deacetylase 3
SIRT6: Sirtuin 6
OPG: Osteoprotegerin
Foxn1: The forehead box protein N1
BMP4: Bone morphogenetic proteins 4
miRNA: MicroRNA
ATI: Acute thymic involution
TLRs: Toll-like receptors
HPA: Hypothalamus-pituitary-adrenal
SASP: The senescence-associated secretory phenotype
EV: Extracellular vesicle
SA-β-gal: Senescence-associated-*β*-galactosidase
Jmjd3: Jumonji domain-containing protein 3
Cbx4: Chromobox homolog 4
Eed: Embryonic ectoderm development
H3K27me3: Trimethylation on histone H3 lysine 27
PcG: Polycomb group
PRC1: Polycomb repressive complexes 1
DD: DNA damage
PVS: Perivascular space
EMT: Epithelial-mesenchymal transition
PPARγ: Peroxisome proliferators-activated receptors
AR: Androgen receptors
ER: Estrogen receptors
iTECs: Induced TECs
Fgf7: Fibroblast growth factor 7
IGF-1: Insulin-like growth factor 1
